# *SerumCovid* database: Description and preliminary analysis of serological COVID-19 diagnosis in healthcare workers

**DOI:** 10.1371/journal.pone.0265016

**Published:** 2022-03-17

**Authors:** Isis Didier Lins, Leonardo Streck Raupp, Caio Bezerra Souto Maior, Felipe Cavalcanti de Barros Felipe, Márcio José das Chagas Moura, João Mateus Marques de Santana, Alexsandro dos Santos, Marcelo Victor de Arruda Freitas, Ramon Nascimento Silva, Ewerton Henrique da Conceição, José Cândido Ferraz, Alice Araújo, Mariana Fernandes, Ana Lisa Gomes

**Affiliations:** 1 CEERMA—Center for Risk Analysis, Reliability Engineering and Environmental Modeling, Universidade Federal de Pernambuco, Recife, Brazil; 2 Department of Production Engineering, Universidade Federal de Pernambuco, Recife, Brazil; 3 Techology Center, Universidade Federal de Pernambuco, Caruaru, Brazil; 4 Vitória Academic Center, Universidade Federal de Pernambuco, Vitória de Santo Antão, Brazil; Government College University Faisalabad, PAKISTAN

## Abstract

Serological databases represent an important source of information to perceive COVID-19 impact on health professionals involved in combating the disease. This paper describes *SerumCovid*, a COVID-19 serological database focused on the diagnosis of health professionals, providing a preliminary analysis to contribute to the understanding of the antibody response to the SARS-CoV-2. The study population comprises 321 samples from 236 healthcare and frontline workers fighting COVID-19 in Vitória de Santo Antão, Brazil. Samples were collected from at least six days of symptoms to more than 100 days. The used immunoenzymatic assays were Euroimmun Anti-SARS-CoV-2 ELISA IgG and IgA. The most common gender in *SerumCovid* is female, while the most common age group is between 30 and 39 years old. However, no statistical differences were observed in either genders or age categories. The most reported symptoms were fatigue, headaches, and myalgia. Still, some subjects presented positive results for IgA after 130 days. Based on a temporal analysis, we have not identified general patterns as subjects presented high and low values of IgA and IgG with different evolution trends. Unexpectedly, for subjects with both serological tests, the outcome of IgA and IgG tests were the same (either positive or negative) for more than 80% of the samples. Therefore, *SerumCovid* helps better understand how COVID-19 affected healthcare and frontline workers, which increases knowledge about the infection and enables direct prevention actions.

## 1 Introduction

The global health crisis established in March 2020 by the Severe Acute Respiratory Syndrome Coronavirus 2 (SARS-CoV-2) mobilized the entire scientific community to better understand and combat the pandemic. In the severe acute respiratory syndrome (SARS) epidemic in 2002–2003, the healthcare workers (HCW) represented the high-risk group of infection with rates around 20% [1]. Nowadays, the current coronavirus disease 2019 (COVID-19) quickly spreads and infects millions of people worldwide [2,3], an emergency in which the HCW conditions again represent a challenge faced by global public health systems [4]. Indeed, HCW have absorbed substantial risks of acquiring COVID-19 due to their care of patients with COVID-19 infection throughout the pandemic [5].

Coronaviruses are a group of enveloped viruses with non-segmented, single-stranded, and positive-sense RNA genomes [6]. Several studies have investigated infection rates and epidemiological and risk factors in HCW worldwide [7,8]. Serology is a promising tool for identifying individuals with a previous infection by detecting antibodies generated in response to SARS-CoV-2 [9]. In fact, antibody tests for COVID-19 have been increasingly deployed to estimate the seroprevalence of antibodies to SARS-CoV-2 [10].

The serological test for the presence of antibodies against SARS-CoV-2 might provide a more accurate estimate of the cumulative prevalence of SARS-CoV-2 infection in a population compared to the viral test, as the antibodies against the virus, in particular IgG, are likely to persist for a more extended period after the viral infection [11]. Gómez-Ochoa *et al*. [12] recently performed a systematic review of SARS-CoV-2 infection among HCW, including 97 studies on eight virtual medical bibliographic bases. The authors estimated an antibody prevalence of 7%, with [4%, 11%] as a 95% confidence interval.

In this context, databases in health are defined as a collection of organized and stored, accessible, and retrievable data. Then, building and providing an open database with data of age group, gender, diagnosis, professions and extracting information from them in the pandemic scenario offers relevant support to elaborate preventive and monitoring actions [13].

Here, we present the descriptive and preliminary analysis performed on *SerumCovid* database, which allows comparison between several HCW subgroups. This paper describes a database gathered in a northeastern city of Brazil from June 2020 to January 2021 focused on diagnosing HCW using tests for IgA and/or IgG antibodies. Subjects who presented suggestive clinical symptoms of respiratory syndromes and underwent clinical evaluation were invited to the study. Additionally, each participant was asked to answer a questionnaire including clinical data.

## 2 Materials and methods

### Population identification

The study population comprises HCW and frontline workers who had suspicious symptoms of COVID-19 or positive laboratory humoral immune response results for viral infection in Vitória de Santo Antão (VSA). VSA, a municipality situated 40 km from Recife (Pernambuco, Brazil), has 139,583 inhabitants and registered 2,550 confirmed COVID-19 cases in 2020 [14]. We consider frontline workers who are not HCW but were fundamental to combat the pandemic (e.g., police officers and researchers). They perform essential functions (e.g., security and healthcare-related) for the functioning of society in the situation of social detachment necessary for the control of the pandemic. In addition, for specific analysis, we separate the HCW category into three main classes: hospital workers (e.g., nurses, doctors), basic healthcare workers (e.g., dentist, psychologist), and laboratory workers (e.g., biomedical, pharmaceutical). The *SerumCovid* dataset used in this paper is provided in the supporting information (S1 File ).

### Serum samples

The samples were initially collected in the Vitória Specialty Center, and then, the whole blood was processed in the Central Laboratory of Vitória. Finally, the frozen serum was sent to the Academic Center of Vitória/UFPE for subsequent serological analysis.

### Antibody research

The Enzyme-Linked Immunosorbent Assays (ELISA) were the Euroimmun (Lübeck, Germany) Anti-SARS-CoV-2 ELISA IgG and IgA assays following the manufacturer’s guidelines with all samples taken in duplicates. The kit targets spike recombinant proteins, Subunit S1, tested in different research groups with varied cohorts and populations. It demonstrated good sensitivity (90%) and specificity (92.5%) to IgA and excellent specificity when combining IgG and IgA (100%) in samples collected after only four days of symptoms [15] despite the test optimization for 30 days of symptoms. The semiquantitative results of the ELISA were analyzed using an electronic spreadsheet to calculate the mean between the duplicates and then the ratio of the absorbance value of the control or the patient’s sample on the absorbance value of the calibrator. The test was considered positive >1.1, negative < 0.9, or inconclusive for values between 0.9 and 1.1. The results were sent individually to each patient by e-mail, and the team clarified any doubts that might have arisen.

### *SerumCovid* adjustments

**Fig 1:**
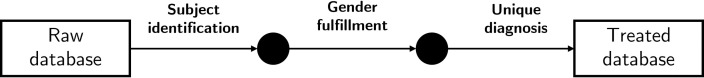
*SerumCovid* initial adjustments.

The initial step in the analysis process is to adjust the entire dataset to handle better a precise statistical analysis (Fig 1). Firstly, because it was a manually inputted database, we checked for (i) double entrances and (ii) same subject entrances with distinct names caused by abbreviations or typos. We verified the ‘CPF’, a Brazilian document for personal identification, as validation. Additionally, we filled the gender missing data according to the subject name.

### Database

*SerumCovid* includes information on 236 different subjects and a total of 321 samples. Each subject presents two groups of data: input information and questionnaires. The input information group refers to personal information (e.g., ‘name’, ‘birth date’, ‘age’, ‘gender’, ‘ethnicity’, ‘education level’, ‘area of work’) as well as objective medical information (e.g., ‘collection date’, ‘ELISA date’, ‘IgA value’, ‘IgA diagnosis’, ‘IgG value’, and ‘IgG diagnosis’). The questionnaires, to which subjects can choose not to respond, include: ‘presence of symptoms’, ‘date of first symptoms’, ‘number of days with symptoms’. Some subjects do not have all fields filled, even for the input information.

**Fig 2:**
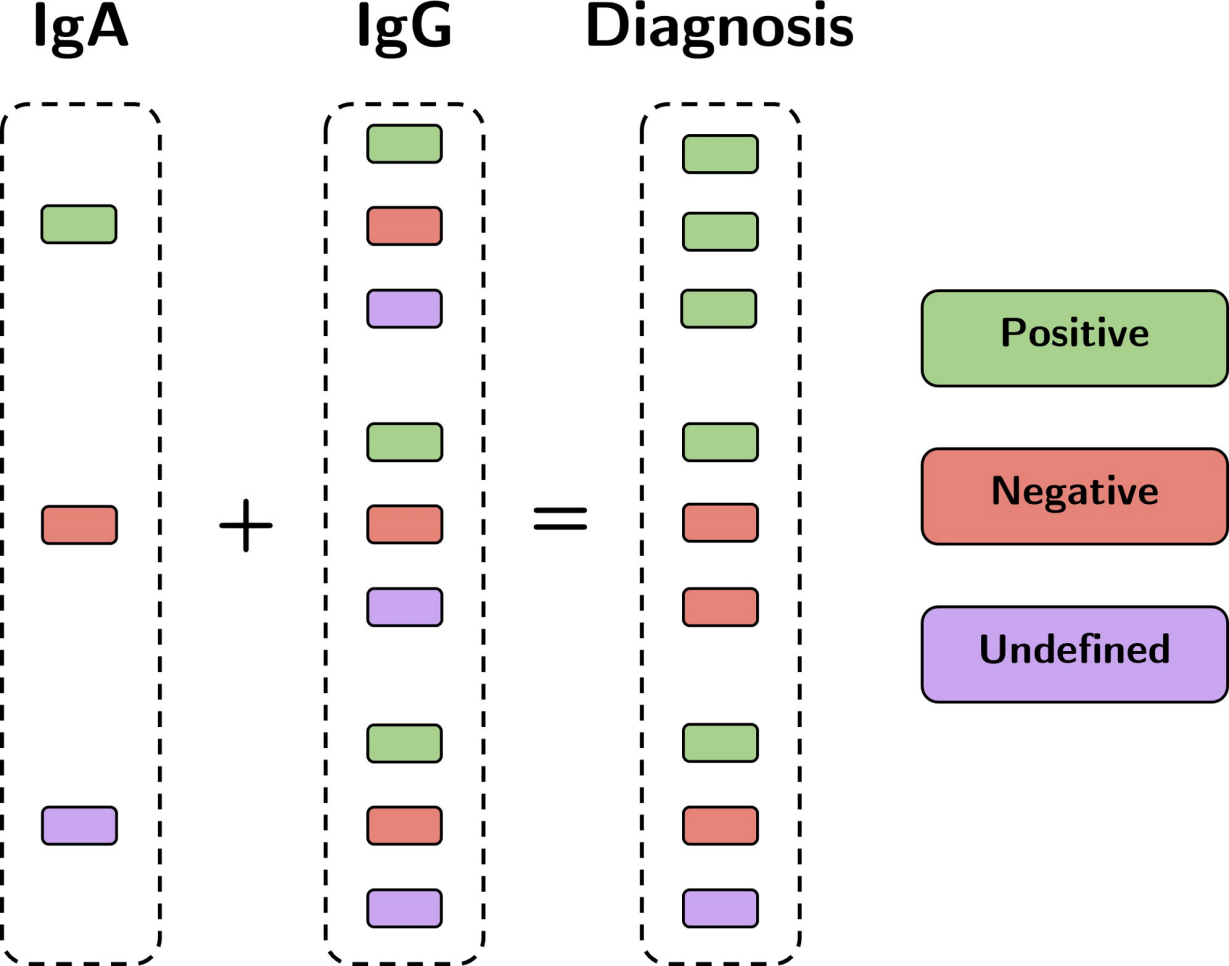
Summarization of all positive combinations of results.

Additionally, as IgA and/or IgG antibodies may have been analyzed, the respective results are presented separated for each antibody. To summarize the individual results in one metric, we consider a positive diagnosis if at least one of the two tests (IgA and IgG) is positive. A negative diagnosis is defined if both tests are negative or if one is negative and the other is undefined (i.e., either inconclusive or not collected). The undefined case, in turn, is when subjects present undefined results in both tests. All possible combinations of IgA and IgG results, and the respective diagnosis, are depicted in Fig 2.

### Statistical analysis

We provided descriptive statistical analyses on the complete database and its stratifications to characterize the profile of subjects. For visualization, we present doughnut charts, pyramid bar charts, bar plots, and violin plots [16]. Specifically, violin charts combine summary statistics and the corresponding continuous probability density curve, providing a more comprehensive visualization of data [17]. Additionally, we used Mann-Whitney [18] and Kruskal-Wallis [19] statistical tests to identify statistical differences depending on the stratification analyzed.

### Ethics

The results presented in this paper compose a larger study with approval of the Ethics Committee on Human Research UFPE—CAV 4.244.984; all participants provided written informed consent.

## 3 Results

### 3.1 Serological profile

Descriptive statistics of IgA and IgG antibodies with *SerumCovid* enable evaluating participants’ serological structure against COVID-19. In this case, the mean and standard deviation of IgA and IgG levels were similar, as shown in Table 1. The information reinforces the robustness of the ELISA test used and the standardization of samples and collection procedures.

**Table 1:** Descriptive analysis of SerumCovid regarding IgA and IgG.

	IgA (N = 142)			IgG (N = 316)		
	Positive	Negative	Inconclusive	Positive	Negative	Inconclusive
Number of samples	55	76	11	97	214	5
Mean	4.083	0.228	0.942	4.831	0.108	0.961
Standard Deviation	2.650	0.172	0.070	2.834	0.144	0.038

**Fig 3:**
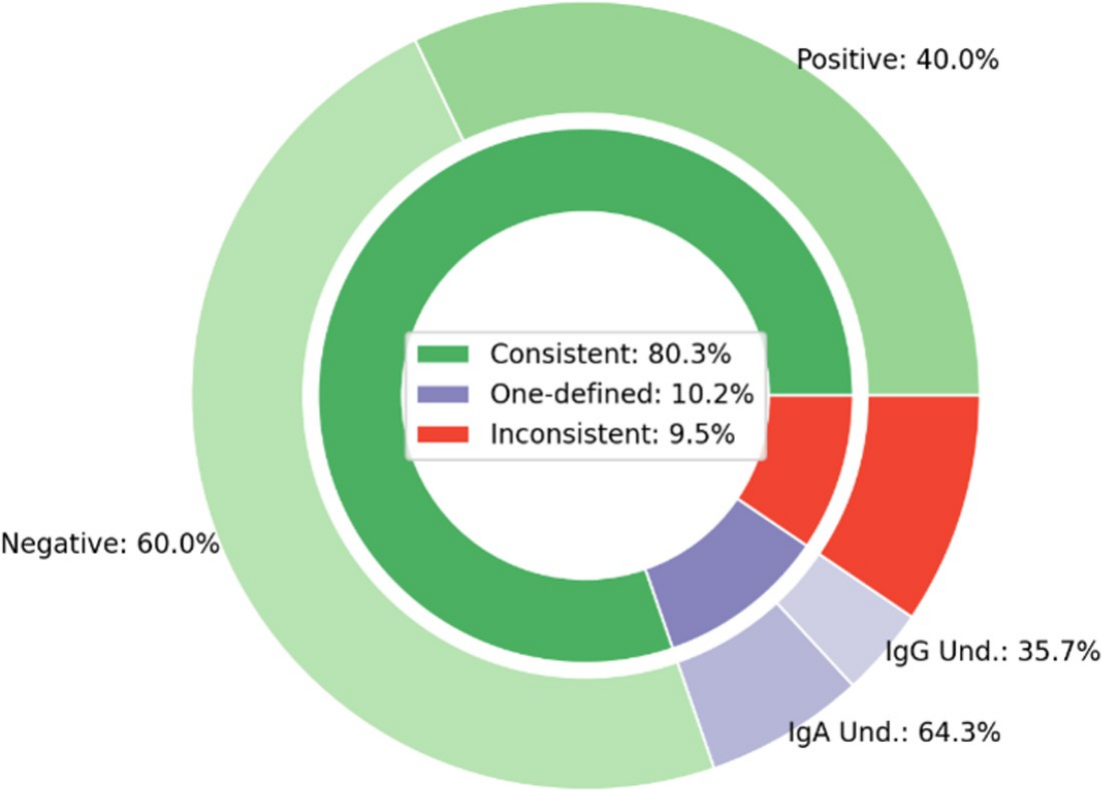
Convergence of results from IgA and IgG tests for the same sample.

From the total of 321 samples, the IgA test was performed in 142 (44.2%) samples, while the IgG test was performed in 316 (98.4%) samples. The intersection comprising 137 (42.7%) samples has IgA and IgG tests information. It is possible to verify the consistency between results (i.e., when both tests were either positive, negative, or undefined). In this case, more than 80% of the IgA and IgG tests were either both positives or negatives. Also, around 10.2% of the samples present only one defined output (positive or negative) while the other is inconclusive. Finally, about 9.5% of data are inconsistent regarding the antibody tests’ results, as IgA and IgG levels indicated opposed outputs (Fig 3).

**Fig 4:**
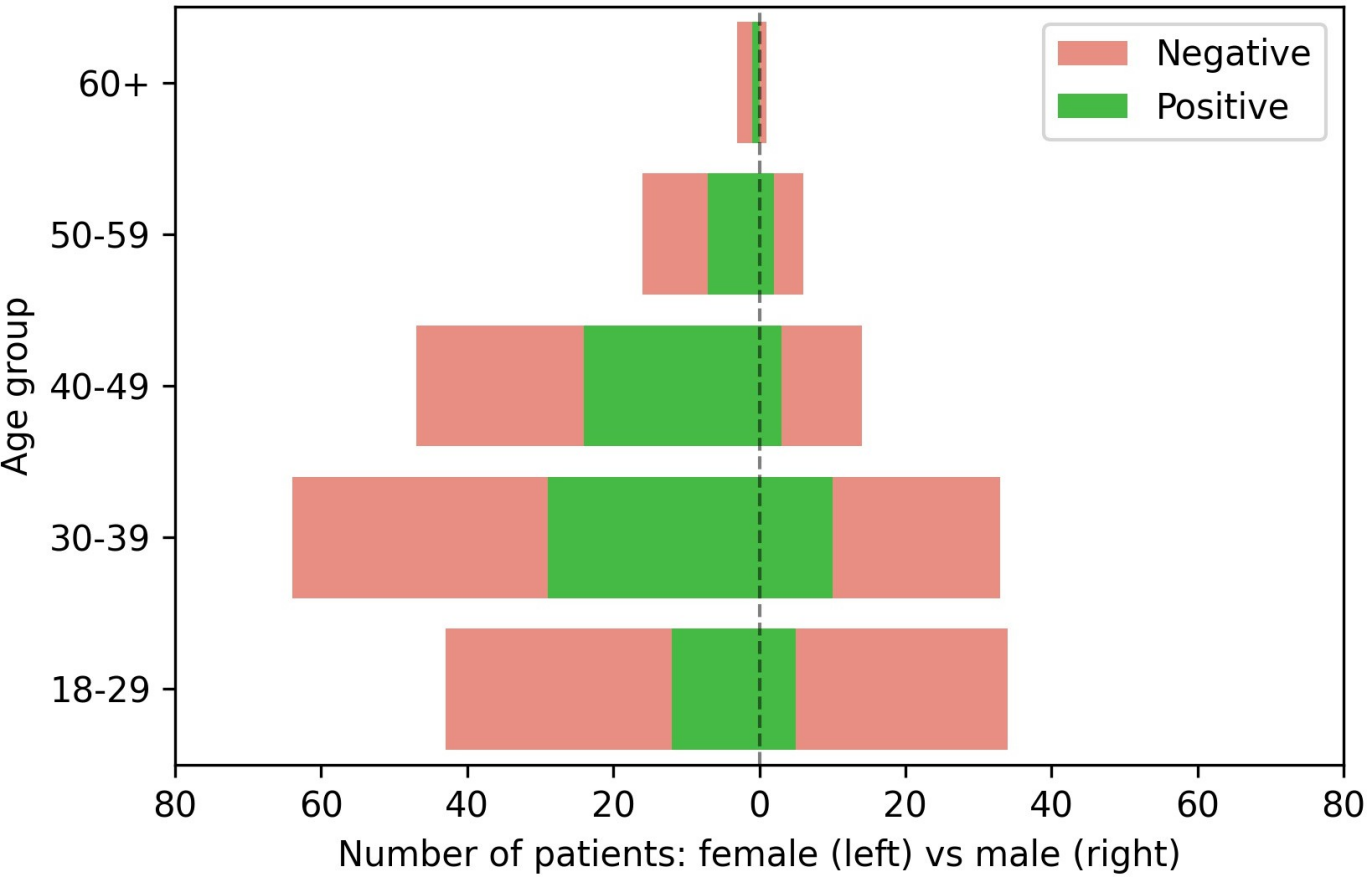
Pyramid combining diagnosis, gender (female: Left; male: Right), and age group.

Adopting the one metric summarization as presented in Fig 2, from the total of 321 samples, three samples are undefined (i.e., both IgA and IgG samples are inconclusive or missing), resulting in a total of 318 samples for which the diagnosis is positive for 108 (33.6%) and negative for 210 (67.4%) samples. In this case, all samples have information about gender (32.1% male and 67.9% female), but age is available in 259 samples. Fig 4 presents a general summary for these samples concerning diagnosis, gender, and age group.

**Fig 5:**
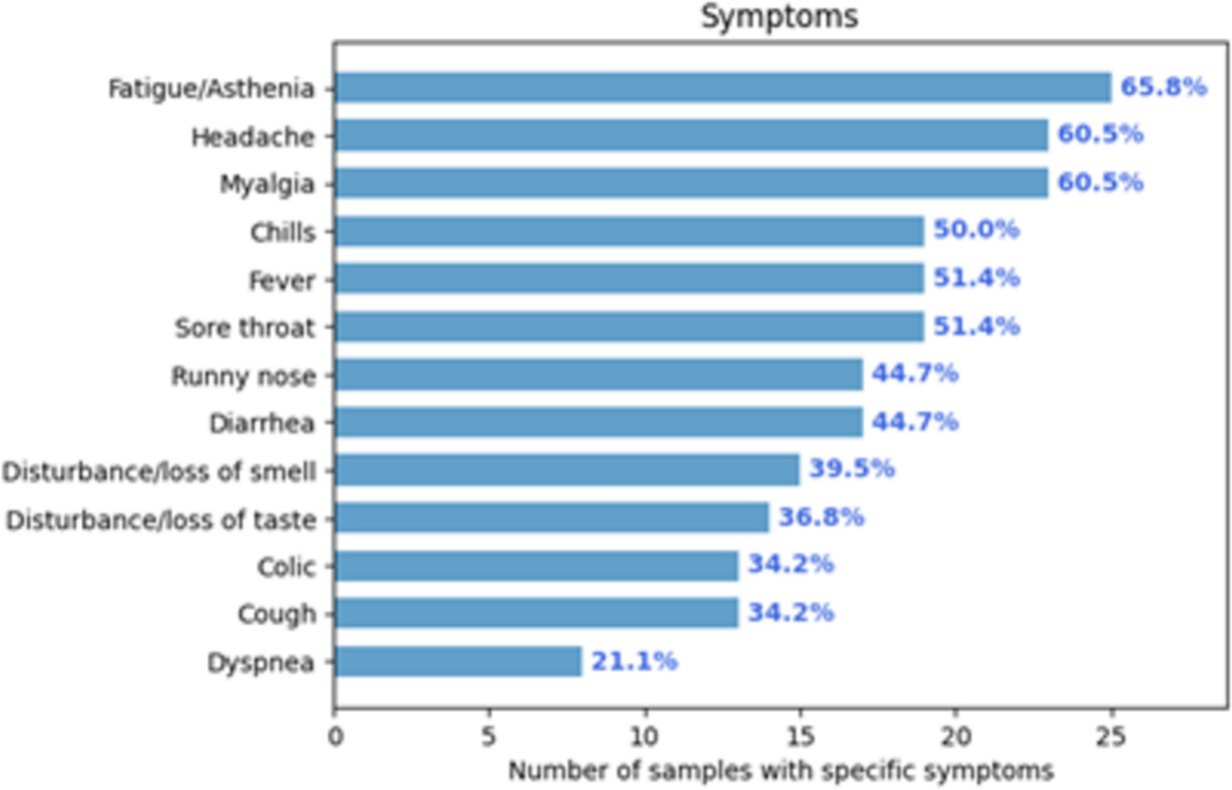
Absolute and relative frequency of samples with a positive diagnosis and specific symptoms.

About symptoms expression, approximately 50.5% of all samples in *SerumCovid* (i.e., 321) are associated with an affirmative answer. However, around 40% of these do not depict specific symptoms due to missing filling. For the remaining samples with at least one specified symptom (~30% of the total), the most frequent for a positive diagnosis were fatigue/asthenia followed by headaches and myalgia (see Fig 5).

**Fig 6:**
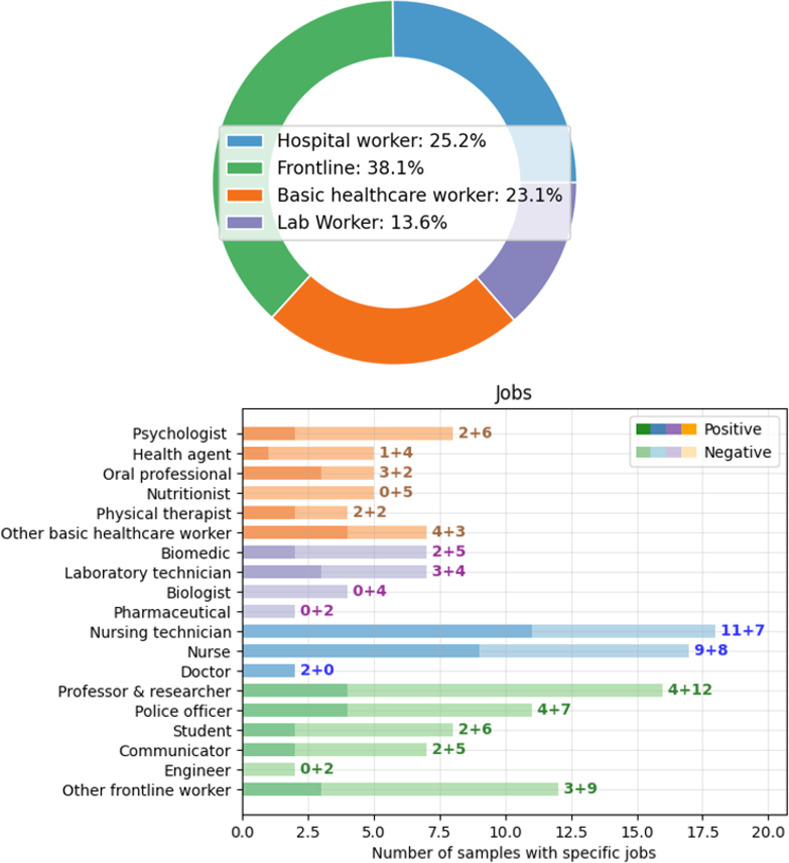
Distribution of categories and jobs on *SerumCovid*. The positive + negative cases are after each job bar.

We also detail the work profile of the subjects analyzed in *SerumCovid*. From the initial 236 subjects, considering the diagnosis definition from Fig 2, two subjects presented undefined classification and, therefore, were excluded from the analysis. For the remaining 234 subjects, 23.0% (i.e., 87 subjects) do not present job information, while 77.0% are associated with distinct jobs for HCW and frontline. As previously mentioned, there were four general categories for HWC: (i) frontline, (ii) hospital workers, (iii) basic healthcare workers, and (iv) laboratory workers (Fig 6). The three main jobs presented in the database are of nursing technician (18 subjects) and nurse (17 subjects), both from (ii), and professors and researchers (16 subjects) from (i). Note there are no outstanding prevalence patterns among the jobs (for example, all two doctors in *SerumCovid* were positive, while all four biologists were negative).

### 3.2 Stratification section

We specifically stratified *SerumCovid* based on the one metric diagnosis (Fig 2) and according to several other characteristics: ‘gender’; ‘age’ (separated in less than 30 years, between 30 and 49 years, or more than or equal to 49 years); ‘time since first symptoms’ (divided into <60 days and ≥ 60 days); and ‘symptoms expression’ (symptomatic or asymptomatic).

#### 3.2.1 Gender

**Fig 7:**
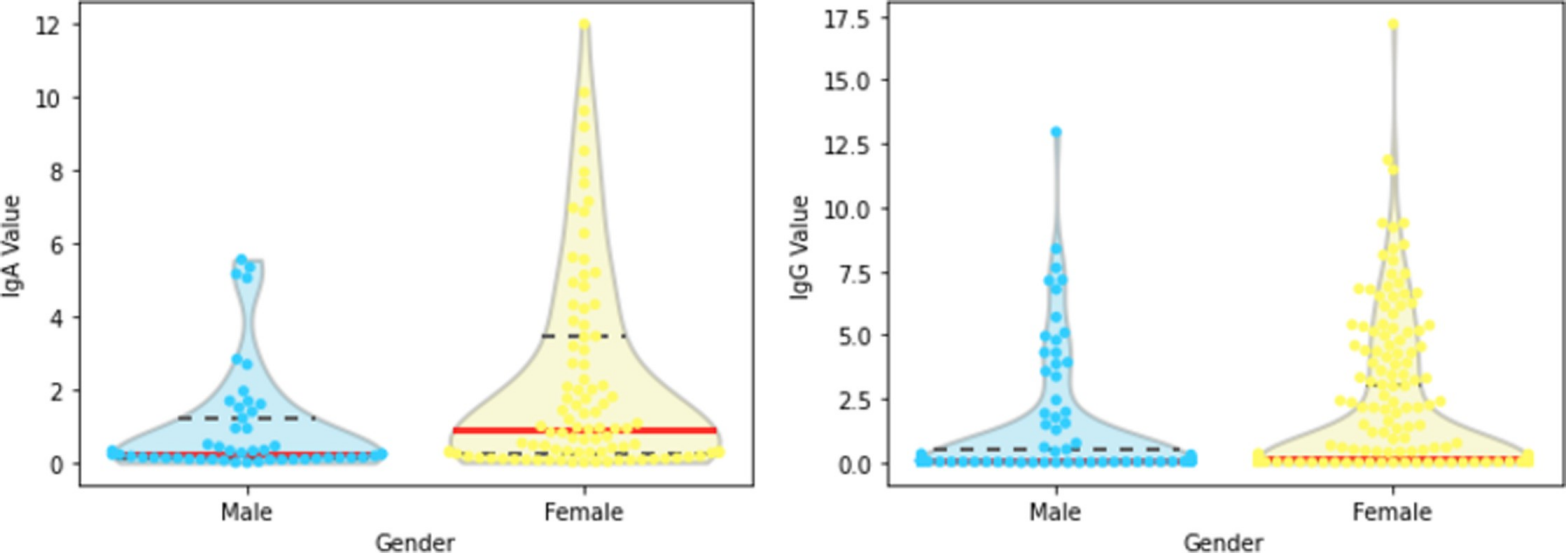
Violin plots with gender and values of (a) IgA and (b) IgG; medians in solid red lines, third quartiles in dashed lines.

Compared to the male group, the female group has a higher mean value and standard deviation for both IgA and IgG (Fig 7). We performed Mann-Whitney statistical test and, considering a significance level of 0.05, the null hypothesis that male and female comes from the same distribution was rejected for both IgA (***p***-value: 0.036) and IgG (***p***-value: 0.007).

#### 3.2.2 Age

**Fig 8:**
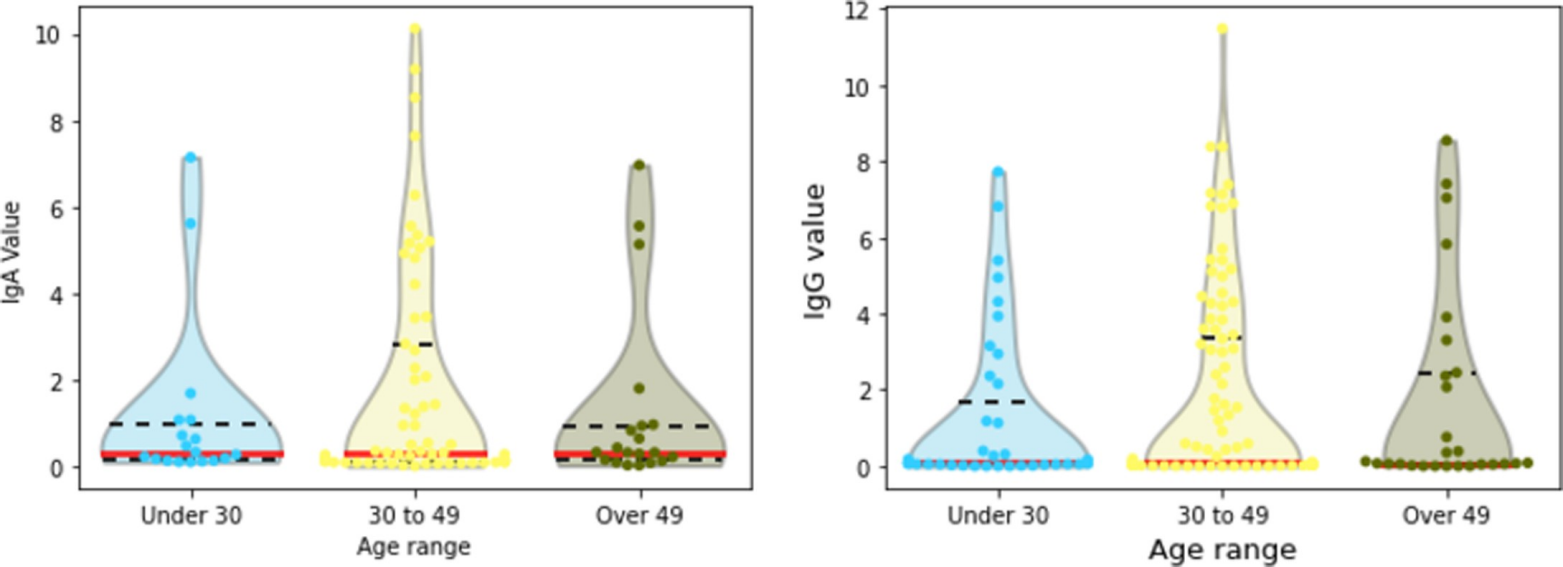
Violin plots of (a) IgA and (b) IgG among age groups; medians in solid red lines, third quartiles in dashed lines.

In this case, most of the data are in the range of 30–49 for both IgA and IgG. The first group (under 30 years) presented the smallest mean and standard deviation for both tests, while a greater value is found for both IgA and IgG for the 30–49 group (Fig 8). In terms of median, none of the three groups seems to present a totally different pattern compared to the others. Indeed, based on the Kruskal-Wallis statistical test and considering a significance level of 0.05, the null hypothesis is not rejected for both IgA (***p***-value: 0.483) and IgG (***p***-value: 0.078).

#### 3.2.3 Time since first symptoms

**Fig 9:**
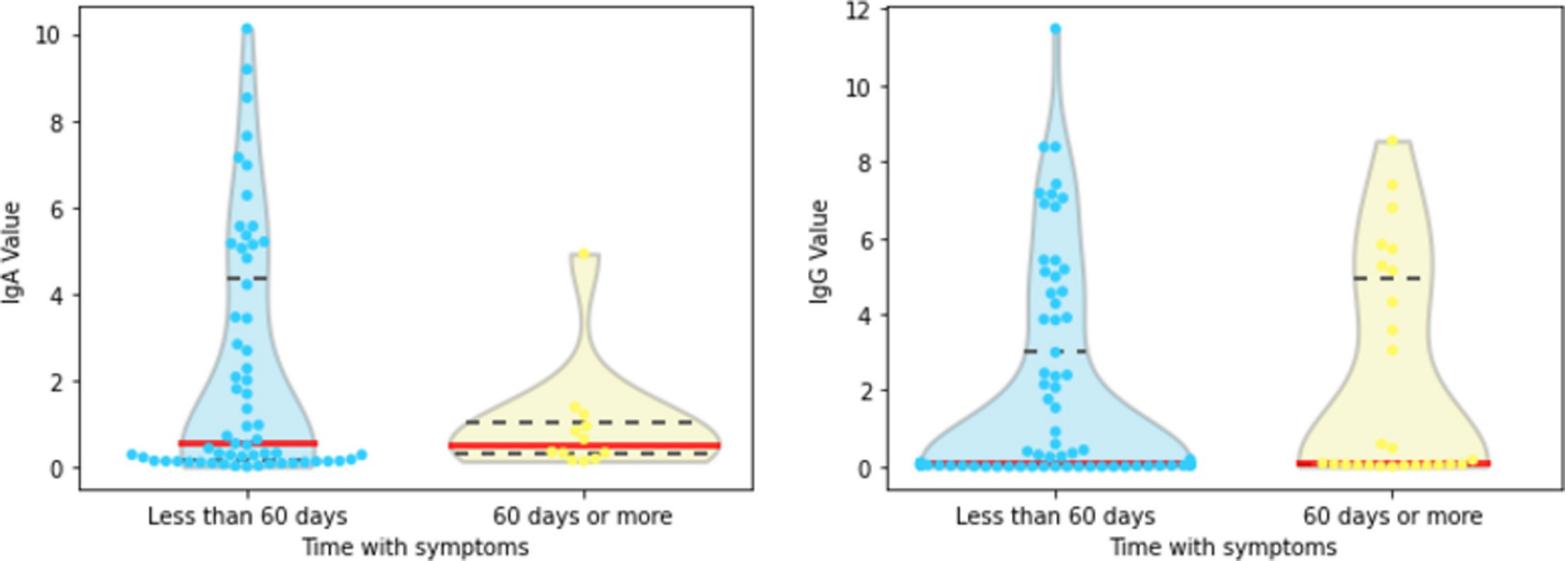
Violin plots of (a) IgA and (b) IgG for time since first symptoms <or ≥ 60 days; medians in solid red lines, third quartiles in dashed lines.

Most of the available data (60 for IgA and 79 for IgG) relates to symptoms in the previous two months (i.e., up to 60 days). Only 12 IgA and 22 IgG samples relate to the first symptoms prior to that (i.e., more than 60 days before). Based on the violin plots from Fig 9, we observe a greater variability for IgA when the time since first symptoms is less than 60 days, which pulls up the mean and standard deviation of IgA values in this group. Nevertheless, as the Mann-Whitney test reported a ***p***-value of 0.396, there is no statistical evidence for shifts in the locations (medians) for IgA values for time since the first symptoms either < or ≥ 60 days. For IgG values, we had the same statistical conclusion (***p***-value of 0.187). However, apart from an extreme observation (IgG: 11.48) in the < 60 days set, we do not observe a variability discrepancy between groups as in the IgA case.

#### 3.2.4. Symptoms expression

**Fig 10:**
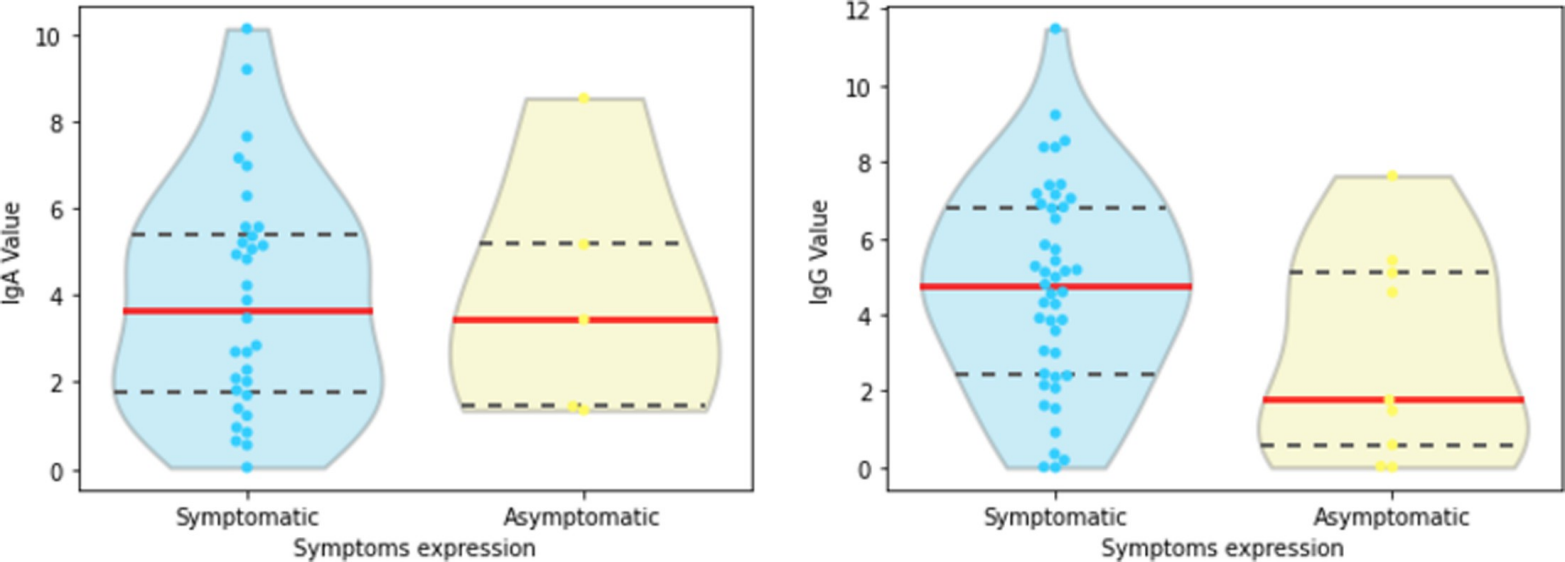
Violin plots of (a) IgA and (b) IgG related to positive results of asymptomatic and symptomatic groups; medians in solid red lines, third quartiles in dashed lines.

Finally, we compared IgA and IgG values related to positive results on symptomatic and asymptomatic groups (Fig 10). For IgA (resp. IgG), we have 32 (resp. 43) samples in the symptomatic set and 5 (resp. 9) in the asymptomatic one. The IgA median values of the two groups do not greatly vary, which is confirmed by the Mann-Whitney test (***p***-value: 0.491). IgG medians, in turn, are visually considerably different in the symptomatic and asymptomatic groups. Still, the statistical test provided ***p*** = 0.05, which is the usual threshold to decide against the equality of medians, possibly due to the small asymptomatic set size.

## 4 Time series analysis

We also performed an analysis of the temporal serological evolution in subjects diagnosed with COVID-19, in accordance with the reasoning depicted in Fig 2, aiming to identify specific patterns. Note from Table 2 that, despite most subjects (i.e., 184) are analyzed once (1 sample collection exactly), 52 subjects are examined two or more times, providing several serological samples. In each collection, a test for either IgA and/or IgG is performed.

**Table 2:** Number of subjects with exactly and at least *n* observations collected.

N° of collections (*n*)	N° of subjects with exact *n* collections	N° of subjects with at least *n* collections
**1**	184	236
**2**	31	52
**3**	15	21
**4**	2	6
**5**	2	4
**6**	2	2

**Fig 11:**
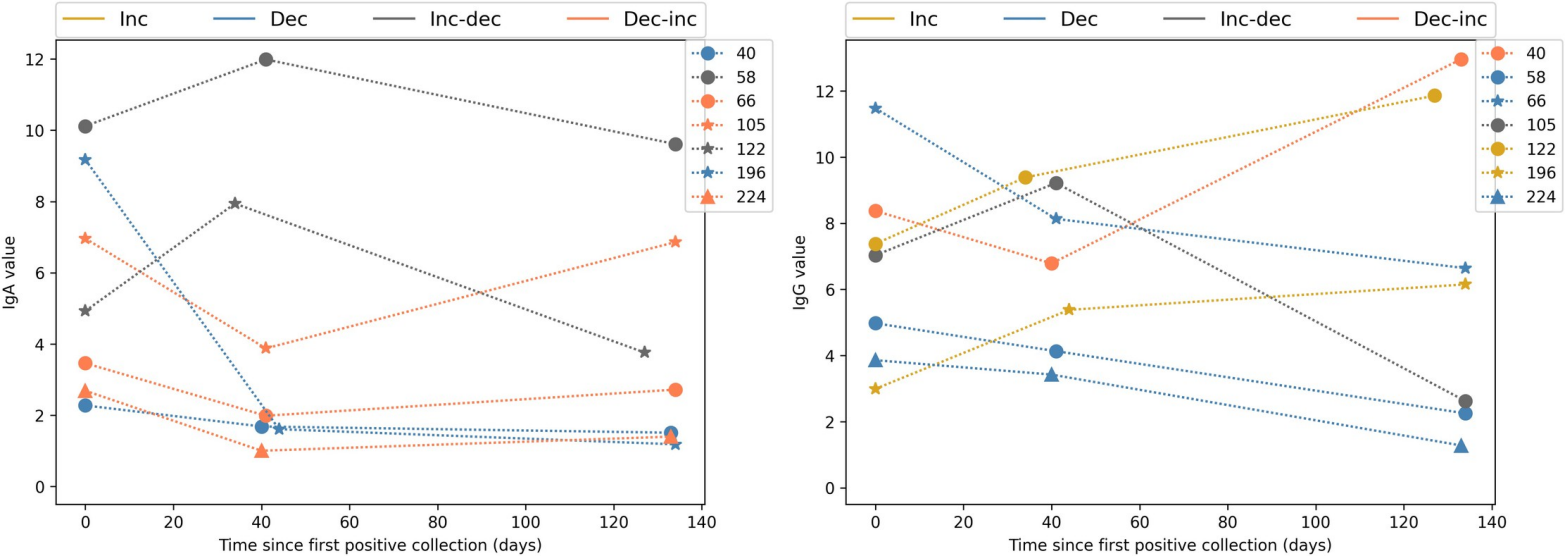
(a) IgA and (b) IgG series of values for subjects with three samples starting with a positive one. Colors denote different behaviors: increasing (yellow), decreasing (blue), increasing-decreasing (gray), decreasing-increasing (orange).

For a richer analysis, we considered subjects with at least three collections (i.e., 21 subjects). This approach resulted in a different number of sequential samples for IgA and/or IgG. However, as previously mentioned, not all samples underwent both IgA and IgG tests. Also, we analyzed the temporal serological evolution after the subject presented a positive result. Therefore, filtering those cases, only seven patients maintained at least three collections starting in a positive one. The exams related to those seven patients are presented separately in Fig 11.

The expected behavior for IgA is to drop sometime after the infection is cleared. However, some subjects have high values of IgA, or even increasing IgA trend, after two months of the positive result (e.g., IDs 58, 66, 105, and 224). Indeed, for IDs 66, 105, and 224, IgA increased, despite an initial decreasing behavior. For IgG, in turn, an opposite behavior is expected: IgG level rises over some months after infection ends. Yet, IgG decreased for four of the seven subjects, even though an initial increase is evidenced for one of them (subject 105). Therefore, we do not identify general patterns, with subjects presenting high and low values of IgA and IgG and with different evolution trends.

## 5 Discussion

The availability of descriptive data directly impacts health and frontline professionals involved in fighting COVID-19 as it permits elaborating care and prevention policies for health conditions monitoring. *SerumCovid* is a database concerning HCW and frontline workers fighting the COVID-19 pandemic in 2020 who had suspicious symptoms, clinical or positive laboratory humoral immune response results for viral infection. Indeed, it has been intensely reported that antibody response to SARS-CoV-2, considering its high relevance for understanding the virus’ clearance, has a crucial role in preventing viral reinfection [20].

Different from Galanis *et al*. [21] who indicated that men would be the most at risk of seropositive for SARS-CoV-2 among HCW and frontline workers, the data in *SerumCovid* has shown that women would be the most exposed to the risk of infection, which is aligned to results reported by Buonafine *et al*. [4]. Also, our observations are consonant with the information provided by the Health Department of the state of Pernambuco in the epidemiological bulletin of July 27, 2021, that 73% of health professionals who had confirmed COVID-19 laboratory positive results were women [14].

In *SerumCovid*, the stratification with the most positive cases was from women aged between 30 and 49 years, similar to the results of Medeiros *et al*. [22]. Indeed, when considering the IgA and IgG values, the highest mean is associated with the range of 30 to 49 years, followed by the over 49 age group, once again in accordance with Medeiros *et al*. [22]. Surprisingly, in the under 30-year-old group, both IgA and IgG values were lower, differently from what was shown by Ward *et al*. [23].

As we used the IgA and IgG values to define a given test result (either positive, negative, or inconclusive), there is a clear distinction in the mean value and standard deviation when comparing positive and negative samples for IgA and IgG (Table 1). This is consonant with the good sensitivity and specificity of the ELISA test with a small number of samples classified as borderline, as reported in Beavis *et al*. [15]. Interestingly, the range and mean values of the IgA and IgG in the serological analyses were similar. Also, we observed no opposite results of both antibodies’ tests (i.e., both positive/negative or one positive/negative and the other undetermined) for 90.5% (Fig 3), which fosters a discussion about whether both tests are indeed imperative. Ma *et al*. [24] presented the importance of detecting IgA between days 4–25 after infection and IgG on days 31–41, and this was reinforced in our data, since between 20 and 60 days after infection, the two tests tended to have the same result. It is not a question of choosing the class of Ig that should be researched, but its importance in the complementarity of understanding the pathogenesis of the disease.

The observation of most frequent symptoms as fatigue/asthenia, headaches, and myalgia, is in agreement with the results of Buonafine *et al*. [4]. These are the most common symptoms observed in COVID-19 in 2020, as clinically there are no specific signs and symptoms of the disease [25]. In *SerumCovid*, the proportion of symptomatic concerning IgA positivity was 86.5%. In comparison, 82.7% was the IgG counterpart. These high figures are expected because an inclusion criterion for the database was subjects with suspect symptoms or positive laboratory humoral immune response results for viral infection.

Here, although the serology analysis concerning days since first symptoms showed that IgA persists for more than three weeks, like the results of Dan *et al*. [26], the mean value decreases compared to samples related to more than 60 days. IgG values, in turn, increase and remain detectable for more than several weeks. However, statistical tests did not show differences between the median value for subjects with less or more than 60 days of symptoms. Thus, based on *SerumCovid*, even without fitting in severe or even moderate cases, the subject classified as mild and even asymptomatic had detectable specific antibodies and continuity of detection of these values. Other researches confirm such information [20,22,26].

We have also performed a temporal analysis as *SerumCovid* contains data from serial collections for specific subjects, ranging from two to six samples per subject. The data suggest the absence or difficulty in identifying a pattern as both IgA and IgG values presented increasing and/or decreasing behavior. It evidences the urgency of monitoring subjects naturally infected or not that may have undergone active immunological sensitization by vaccines [20].

The data presented here are of great relevance. They provide municipal and state management with valuable information to guide policies for monitoring HCW and frontline on their return to work. Indeed, our analysis is relevant as only clinical evaluation in suspected cases, and rapid tests for antibodies’ detection were previously offered to HCW and frontline of VSA municipality. The possibility of performing serological tests of high specificity and sensitivity allowed a more assertive descriptive profile and follow-up of the serological level of COVID-19 infection. It also aided the identification of risks and the necessary measures to mitigate when caring for COVID-19 patients in the basic health units and centers of high complexity in VSA. Our constant dialogue with the municipal authorities favors the development and implementation of policies based on serological evidence of the local HCW and frontline professionals.

Additionally, the collected data serve as a basis for other studies, some already underway by our research group. Our efforts are directed to (i) the monitoring of infection rates among HCW and frontline workers, (ii) the understanding of immunological performance after vaccination, (iii) the analysis of patient life quality after COVID-19 infection, and (iv) the identification of which variables in this database would be more impactful to develop the disease by computational modeling.

The findings in this report are subject to at least three limitations. First, it was not possible to test subjects with molecular RNA SARS-CoV-2, which would be more reliable tests for diagnosis [27]. Second, not all subjects had IgA tests because of funding limitations, restricting a more comprehensive evaluation. Finally, as the database information is from people’s spontaneous responses and manually inputted, several fields were not filled in, which also reduces a broader analysis.

*SerumCovid* is being used as a data source for monitoring a group of subjects for a longer time, including serological dosage after vaccination. These results will be released as soon as the minimum follow-up time is completed. It may provide data that will be important for targeting research and political decision-making to impact the population’s health positively.

## Supporting information

S1 File (XLSX)
